# Inhaled Anesthetics: Environmental Role, Occupational Risk, and Clinical Use

**DOI:** 10.3390/jcm10061306

**Published:** 2021-03-22

**Authors:** Mariana Gaya da Costa, Alain F. Kalmar, Michel M. R. F. Struys

**Affiliations:** 1 Department of Anesthesiology, University of Groningen, University Medical Center Groningen, 9713GZ Groningen, The Netherlands; m.m.r.f.struys@umcg.nl; 2 Department of Anesthesia and Intensive Care Medicine, Maria Middelares Hospital, 9000 Ghent, Belgium; alainkalmar@gmail.com; 3 Department of Basic and Applied Medical Sciences, Ghent University, 9000 Ghent, Belgium

**Keywords:** inhaled anesthetics, environment, climate change, occupational health, patient risk

## Abstract

Inhaled anesthetics have been in clinical use for over 150 years and are still commonly used in daily practice. The initial view of inhaled anesthetics as indispensable for general anesthesia has evolved during the years and, currently, its general use has even been questioned. Beyond the traditional risks inherent to any drug in use, inhaled anesthetics are exceptionally strong greenhouse gases (GHG) and may pose considerable occupational risks. This emphasizes the importance of evaluating and considering its use in clinical practices. Despite the overwhelming scientific evidence of worsening climate changes, control measures are very slowly implemented. Therefore, it is the responsibility of all society sectors, including the health sector to maximally decrease GHG emissions where possible. Within the field of anesthesia, the potential to reduce GHG emissions can be briefly summarized as follows: Stop or avoid the use of nitrous oxide (N_2_O) and desflurane, consider the use of total intravenous or local-regional anesthesia, invest in the development of new technologies to minimize volatile anesthetics consumption, scavenging systems, and destruction of waste gas. The improved and sustained awareness of the medical community regarding the climate impact of inhaled anesthetics is mandatory to bring change in the current practice.

## 1. Introduction

Inhaled anesthetics have been in clinical use since 1844 [1]. Their discovery was a landmark as the first form of general anesthesia. Over the years, improved inhaled anesthetics were developed and implemented in clinical practice, while older drugs were abandoned due to toxic effects [2]. Unlike other drugs in clinical use, inhaled anesthetics carry a specific occupational risk for the health care workers due to their volatile nature. Inhaled anesthetics are highly inert molecules, resulting in minimal to absent biotransformation—and thus virtually no production of toxic metabolites. This unique chemical property, however, also results in exceptional atmospheric stability, causing these powerful greenhouse gases to have long-lasting ecological effects, requiring special attention from researchers, policymakers, and society in general.

A 2019 *The Lancet* article from an international collaboration dedicated to studying climate change on health stated that, based on current indicators, climate changes are occurring faster than government responses [3]. In order to revert the actual climate crisis scenario, new approaches are needed. Given the excessive ecological effects of volatile anesthetics, anesthesia bears an important responsibility here. However, an inclusive study reporting the (dis) advantages for the patient versus occupational risks and environmental effects is warranted to have a well-considered analysis of the possible clinical impacts of any changes in anesthesia practices. Therefore, this review recounts the results of the available scientific literature and interpretation of data related to the inhaled anesthetics from three different perspectives:

The first perspective is the environmental effect of inhaled anesthesia, starting with the current climate crisis, presenting the link between climate change and health, showing the evidence for the role of inhaled anesthetics, and suggesting ideas for improvements.

The second perspective is from an occupational point of view, which investigates the risks that exposure to inhaled anesthetics during the work journey can bring to healthcare workers.

The third perspective is from the clinical side, discussing potential benefits or harm from using inhaled anesthetics to the patient in different clinical contexts.

The clinical discussions presented in this review focus on evaluating the need for inhaled anesthetics. They do not function as a pro–con comparison between the use of total intravenous anesthesia (TIVA) and inhaled anesthesia, but rather briefly show the available evidence from different areas where inhaled anesthetics are used.

## 2. Inhaled Anesthetics and the Environment

### 2.1. Global Warming

The average global temperature is rising rapidly, a phenomenon that is causing dramatic climate changes. The last 5 years have been the hottest since the industrial era [4]. Between 2030 and 2052 an average increase of 1.5 °C or higher in the global temperature is expected when compared with the pre-industrial levels [5].

The temperature of the earth depends on the balance between the radiation energy received and emitted by the planet. The planet receives radiation energy from the sun. A part of the solar radiation is directly reflected back into space, a part is absorbed by the atmosphere and the surfaces—land and ocean—and ultimately emitted back to space. The difference between the radiation received and the radiation emitted back is called radiative forcing. The wavelengths of the incoming solar energy is largely between 0.1–2.0 µm, whereas the wavelength of the outgoing energy from the earth is in the much longer wavelength range of infrared light [6]. Some specific gases in the atmosphere absorb the outgoing radiation within this wavelength range. The energy is subsequently converted into heat, which helps keep the lower layer of the atmosphere warmer. Gases with this ability to absorb infrared light are the so-called greenhouse gases (GHGs). The most important GHGs are CO_2_, methane (CH_4_), water vapor, nitrous oxide (N_2_O), and fluorocarbons. The phenomenon of trapping heat in the atmosphere is known as the greenhouse effect, which is a natural and necessary process that enables life on earth. However, in the last century, the human activity has rapidly increased the release of GHGs in the atmosphere, resulting in the excessive greenhouse effect. This phenomenon, called global warming, has led to an increase in average temperatures despite relatively small variations in the received solar energy [7].

### 2.2. Climate Change, Health Impacts, and Challenges for Reducing Emission of GHG

Considering the looming climate crisis, multiple effects on human health are expected. The World Health Organization considers climate change the greatest threat to the global human health [8]. The impact of climate change on health can be direct or indirect [9]. Directly, heat or cold waves will lead to higher morbidity and mortality, especially in a high-risk population such as children and elders, while floods and storms are likely to increase the rate of transmissible diseases. Indirectly, health problems are anticipated resulting from environmental modifications such as changing patterns of disease-carrying vectors, similar to mosquitos or ticks, resulting in an increase in water-borne diseases owing to higher temperatures and increasing rainfall [9].

In 2018, *The Lancet* reported and summarized the direct and indirect effects of climate change [10]. They concluded that climate change is expected to influence the environment, leading to increases in cardiovascular and pulmonary disease, undernutrition, diarrhea, and vector-borne disease, etc. In addition, social consequences from this scenario would be the loss of habitation, mass migration, and poverty and could also result in violent conflicts.

The human activity is the principal trigger of the current warming of the planet. In 1997, the Kyoto Protocol was an important step from the international community in discussing and setting limitations and targeted emission reductions. The 2015 Paris agreement, which included more nations, established the goal of strengthening efforts to limit global warming to 2.0 °C or even a more desirable 1.5 °C. To achieve these goals, an extensive reduction in greenhouse gas emissions is necessary [10]. While the biggest changes need to happen at the governmental and societal levels, anesthesiologists may make a significant difference as individuals.

### 2.3. The Carbon Footprint of the Healthcare Sector

The healthcare system itself generates a considerable amount of GHGs, which has, so far, received little attention. Importantly, GHGs other than CO_2_ contribute particularly strongly to the total GHG emissions of the healthcare industry [11]. Therefore, it is important that GHG emissions coming from the healthcare sector are taken into account in the assessments on health and climate [10]. Pichler et al. estimated an average healthcare carbon footprint of 5.5% (3.3–8.1%) from the total percentage of the national footprint in 36 countries between 2000 and 2014 [12]. Likewise, the carbon footprint of the healthcare sector is estimated to correspond to 8% of the total CO_2_ emissions in the Netherlands, and 7% in the United States of America [12,13]. If global healthcare was considered a country, it would be the fifth highest with regards to GHG emissions [11].

### 2.4. Quantification of the Warming Effect of Pollutants

**Figure 1:**
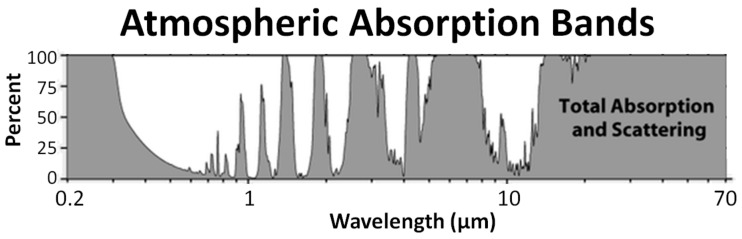
Atmospheric absorption bands. Modified from an image obtained from the Wikimedia website, available under a CC-BY-SA 3.0 license, and included in this review on this basis.

A commonly used measure to quantify and compare the warming effect of each gas is called global warming potential (GWP). The GWP of each gas is mainly defined by its half-life in the atmosphere and its spectrum-specific radiation absorption capacity. Obviously, a gas that remains in the atmosphere for decades to centuries will have the ability to trap much more heat, compared to a gas that is eliminated within days after release. Second, while solar radiation mainly consists of waves in the range of visible, ultraviolet, and near infrared light, with wavelengths between 100 nm (UV) and 4 µm (near-IR)), the earth emits its heat in the range of longer wavelengths. Within the electromagnetic spectrum of emitted energy by the earth, most of the wavelengths are unavailable for cooling since natural GHGs (mainly water, CO_2_, and N_2_O) absorb virtually every photon of many infrared wavelengths emitted by the earth. Only small “windows” remain for certain wavelengths to escape to space. A gas with an absorption at the wavelengths of the “open windows” (white zones in Figure 1) will induce more additional heat-trapping effects compared with a gas with absorption at wavelengths for which the atmosphere is already impenetrable (grey zones in Figure 1). As such, the additional absorption by a pollutant is highly variable depending on its absorption spectrum.

A previous IPCC report described the dedicated method for GWP calculations [14]. CO_2_ is used as a reference for all GWP calculations_,_ and by definition has a GWP of 1. Consequently, the GWP of the other gases is quantified relative to the warming effect of CO_2_. To include the relevant time frame of heat-trapping, the time range reference is expressed, with the denotation GWP_(t)_. The most common time periods used for the GWP estimates are 20, 100 or 500 years, with GWP_(100)_ being the most common and the reference used in the Kyoto Protocol. For example, if a gas has a GWP_(100)_ of 298, it means that 1 kg of this gas captures the same amount of heat over a period of 100 years as 298 kg of CO_2_.

### 2.5. Contribution from Inhaled Anesthetics

There are important differences in the global warming effects of different volatile anesthetics. First, depending on its specific molecular structure the atmospheric half-life varies from a few years to more than a century. Second, the intrinsic ability of the different volatiles to absorb infrared wavelengths in the atmospheric windows varies within orders of magnitude. Third, the amount of agent used in the inhaled gas mixture varies between a few percent to 70%. Notoriously, 1-h of anesthesia performed with desflurane contributes 26 times more to global warming than the equivalent anesthesia performed with sevoflurane. Likewise, the addition of N_2_O increases the global warming impact of sevoflurane-based anesthesia with 590% [15].

Whenever used in clinical conditions, virtually the total amount of gases used during any procedure is eventually discarded to the atmosphere [16]. In the atmosphere, the wavelength of infrared absorption by the inhaled anesthetics overlaps the range of the atmospheric window [17]. Therefore, inhaled anesthetics absorb the radiation and re-emit it to the earth, contributing to the global warming process. Unfortunately, the majority of these gases also last for years to centuries in the atmosphere, intensifying their global warming effect. In 1999, the global warming impact of inhaled anesthetics was estimated as 0.03% of the total global warming and more recently 0.6% [11,18]. As a consequence of increasing the use and long half-life values, global atmospheric levels of inhaled anesthetics are steadily increasing [19]. Projections about the inhaled anesthetic market suggest that the total inhaled anesthetic volume in use, and consequently in the atmosphere will have increased 4.0% between 2015 and 2024 [20].

A clearer understanding about the environmental impact of inhaled anesthetics can be achieved by comparing it to the emission of CO_2_ during a single car trip. The use of inhaled anesthetics for 1 h in their commonly used concentrations of 1 MAC and a FGF of 1 L·min^−1^ has the CO_2_ equivalency as a car trip of 6.5 km for sevoflurane, 14 km for isoflurane, 95 km for nitrous oxide, and 320 km for desflurane [21].

Currently, the most commonly used inhaled anesthetic agents are the hydrofluorocarbons sevoflurane and desflurane, the chlorofluorocarbon isoflurane, and N_2_O. The Montreal Protocol, adopted to control chlorofluorocarbons, was signed in 1987. While it was amended in 2016 to also reduce the use of hydrofluorocarbons, owing to the medical relevance of inhaled anesthetics, these substances were systematically excluded [22].

#### 2.5.1. Desflurane

Desflurane is the volatile anesthetic with the highest GWP_100_ of 2540 and an atmospheric lifetime of 14 years [23]. Worldwide, the total estimated equivalent release of total CO_2_ (tCO_2_) by hydrofluorocarbon anesthetics was approximately 3 million metric tons, with desflurane accounting for 2.5 million [19]. MacNeill et al. demonstrated the impact of desflurane use in clinical practice in a well-designed report, in which huge—10 fold—disparities in GHG emissions between hospitals could largely be accounted to the use of desflurane in comparison with less harmful gases [24].

#### 2.5.2. Sevoflurane

Sevoflurane has GWP_100_ of 130 and an atmospheric lifetime of 1.1 year [23]. In addition, since with sevoflurane an end-tidal value of around 2% is targeted, compared to 6% with desflurane, the actual amount of emitted chemicals for 1 h of anesthesia is even three times higher with the latter.

#### 2.5.3. Isoflurane

Isoflurane has a GWP_100_ of 510, and an atmospheric lifetime of 3.2 years [23]. Importantly, isoflurane contains a chlorine atom and consequently induces ozone destruction. Hence, it should be discouraged in favor of sevoflurane [19].

#### 2.5.4. N_2_O

N_2_O has a very weak anesthetic potency and is therefore administered in very high doses (60% of inhaled gas mixture versus 2% for sevoflurane). Furthermore, it is commonly combined with other anesthetics, as a carrier gas, since it is not potent enough to lead to a full anesthetic state alone. Although the concomitant use of N_2_O theoretically permits a reduction in the amount of the other inhaled anesthetic in use, N_2_O itself has a GWP_100_ of 298 and an atmospheric lifetime of a staggering 114 years [23]. Due to its extremely long atmospheric lifetime and the high consumption volumes, N_2_O becomes a major contributor to global warming.

#### 2.5.5. The Carbon Footprint of Anesthetics

Although the impact of inhaled anesthetics is lower compared with other GHGs such as methane and black carbon, the current projections for an increase in the use of inhaled anesthetics represent a significant burden on the future greenhouse effect [25]. The GWP of an anesthetic is crucial in establishing its carbon footprint, but there are many other factors that must be considered: CO_2_ emissions during manufacturing, transport, delivery, and even during a possible destruction of each anesthetic. Therefore, the life cycle assessment is the methodology used to estimate the environmental impact of all the processes involved from creation to destruction of a determined product [26]. Accordingly, one study assessed the life cycle GHG emissions of inhaled anesthetic drugs and compared it to propofol [27]. Desflurane showed the greatest life cycle GHG emission, 15 and 20 times higher compared with isoflurane and sevoflurane, respectively. Undeniably, propofol showed by far the lowest total GHG emission. Even when considering emissions in the production processes and additional waste, the GHG release of propofol is about four orders of magnitude lower compared with those of volatile anesthetics [27].

### 2.6. Flow Rate of Inhaled Anesthetics

The environmental impact from inhaled anesthetics depends not only on the inherent characteristics of each gas but also on the amount used. To ensure adequate general anesthesia, the administered dose relates to the minimum alveolar concentration (MAC), which is 2% for sevoflurane, compared to 6% for desflurane. Although there is no globally accepted definition of low-flow anesthesia, conventionally a fresh gas flow (FGF) of ≤1 L·min^−1^ is considered low-flow anesthesia [28]. Contemporary ventilators permit even much lower FGF of 0.1 L·min^−1^, allowing maximal gas recirculation and reducing anesthetic waste. In addition to the less consumption of inhaled anesthetic, resulting in reduced costs and environmental impact, other benefits of minimal FGF include conservation of temperature and humidity in the system [29]. An investment in modern ventilation systems as such may simultaneously enable environmental and financial savings and improved patient safety [28].

### 2.7. Costs

Inhaled anesthetics represent an important part of the costs of the anesthesia department. The costs of any procedure are dependent upon the type of anesthetic used, the targeted patient concentration of volatile anesthetic, and the FGF [30]. The estimated cost at 1 L/min for desflurane is USD 12.96, whereas for isoflurane and sevoflurane it is USD 0.52 and 6.05, respectively [31]. In practical terms, desflurane is the most expensive inhaled anesthetic.

Different experiences with cost savings have already been reported. By discouraging the use of desflurane in favor of sevoflurane, an estimated saving of more than USD 100,000 over 1 year was reported [30]. Similarly, in another service, after a recall from the manufacturer, desflurane was no longer available in the operating room unless the anesthesiologist requested it. With this intervention, there was an absolute reduction of 25.2% in the use of desflurane, an increase of 2.6% in the use of sevoflurane, and an increase of 17.2% in the use of isoflurane. These modifications also resulted in a saving of more than USD 100,000 per year [32].

### 2.8. Suggestions for Mitigating the Impact of Inhaled Anesthetics on Global Warming

A much more sustainable anesthesia practice can easily be achieved with a conscious decision-making process for the choice of and use of inhaled anesthesia. At the moment, most anesthesiologists in operating rooms are still largely unaware of the climate impact that they can produce or prevent. The current curriculum from specialization programs to form anesthesiologists, anesthesiologist assistants, and nurses hardly ever includes scientific information about the environmental impact of inhaled anesthetics. Therefore, increasing the awareness of healthcare professionals may represent an easy and accessible step towards greener practices. Likewise, (online) educational programs towards active professionals may be organized as part of continuing medical education. Positive experiences with the education of the anesthesia team regarding the use of inhaled anesthetics were already reported [33]. Implementing an initial lecture on the theme, followed by a continuous online education, reduced the use of desflurane by 64% over a period of 3 years, resulting in an estimated cost savings of USD 25,000 per month. Remarkably, the motivational factor to implement changes in clinical practice was greater when the environmental impact rather than monetary savings was the focus [33].

Practical adjustments in daily clinical practice may also contribute to mitigate GHG emissions. Decreasing the FGF of an inhaled anesthetic is an easy and achievable approach that can tackle two important issues: The ecological impact of an inhaled anesthetic and the costs of its consumption. Furthermore, the incentive to implement closed-circuit systems is an easy strategy to dramatically reduce inhaled anesthetic waste.

Considering the high pollutant effect of desflurane and the low clinical difference compared with less-polluting alternatives, it should be replaced by more eco-friendly options. Likewise, considering its high pollutant impact and particularly long atmospheric lifetime, N_2_O should be avoided as a carrier gas in clinical practice (when possible). The American Association of Anesthesiologists recently started a campaign encouraging hospitals to reduce the use of desflurane and N_2_O as well as inhaled anesthetic by 50%. These ideas should be stimulated and applied on a global scale [34].

Improving and investing in the development of scavenging systems could also mitigate the environmental effects of inhaled anesthetics. In recent years, new scavenging systems have been proposed to adsorb any waste gases on activated carbon or zeolite for subsequent reuse or destruction [35]. However, the safety and usability of these new systems, as well as the cost-effectiveness, have not yet been demonstrated [36]. An important consideration in this context is that N_2_O is not captured by these systems. A recently proposed technology envisions the direct destruction of inhaled anesthetics through gas-phase photochemistry [37]. Although at this stage only results of prototype experiments are available, this technology would enable a convenient add-on destructor for turning the volatile anesthetics into harmless gases, which can subsequently be eliminated through the existing exhaust system. In Sweden, the use of different techniques to destroy N_2_O is already practiced and has also shown a beneficial cost effect when compared to other ways to reduce GHG emissions [38].

Xenon is a noble gas, which, in most respects, is an ideal substitute for conventional volatile anesthetics. Contrary to the other inhaled anesthetics, xenon is not a GHG and does not cause environmental damage. Xenon is a trace gas in the earth’s atmosphere (0.0000087%) [39]. Industrial production entails fractional distillation of ambient air. While xenon is absolutely environmentally harmless, its production is very costly and energy-intensive and may, depending on the energy source, therefore also release considerable amounts of GHGs [40]. Research in new technology to reduce the energy requirement and cost of xenon production may open new possibilities in the future as an environmentally benign volatile anesthetic.

A simple solution to avoid the use of inhaled anesthetics is to give preference for TIVA. Through the life cycle assessment, the carbon footprint of intravenous anesthesia is four orders of magnitude smaller compared with using desflurane [27]. Alternatively, in procedures where general anesthesia is not required, the use of local-regional anesthesia is an option.

## 3. Occupational Risks of Inhaled Anesthetics

Waste anesthetic gases (WAGs) correspond to the small amount of gas that leaks from the system in the operating room or that is exhaled by the patient in the recovery unit [41]. During each procedure using inhaled anesthetics, many healthcare workers, including anesthesiologists and nurses, are exposed to WAGs [41]. Although still uncertain, the effects of short or chronic exposure to WAGs can vary from headache, dizziness, and fatigue up to DNA damage [41].

### 3.1. Threshold of Anesthetics in the Workplace

Health surveillance at the workplace is fundamental to prevent risks to professional activity. Promoting occupational health is the responsibility of employers and employees and it is enforced by government regulations [42]. Policies regarding the use of inhaled anesthetics vary among countries. The first regulations were established in the USA, in 1977, when the National Institute for Occupational Safety and Health (NIOSH) determined a threshold of 25 ppm of nitrous oxide (N_2_O) measured as a time-weighted average (TWA) during the administration of the drug, and 2 ppm of the other volatile anesthetics [43]. Others have assigned a threshold of 50 ppm for N_2_O for an 8-h working day [2]. The European Union (EU) uses its own threshold values and exposure limits, which tend to be higher than the North American. Huge discrepancies in threshold limits remain, for example, isoflurane threshold levels vary from a maximum of 5 ppm in Denmark up to 50 ppm in Spain. In the United Kingdom (UK), the maximum levels permitted per anesthetic in an 8-h TWA including 100 ppm for N_2_O, 50 ppm for isoflurane, and 10 ppm for halothane, but there are no defined values for sevoflurane or desflurane. In general, considering that absolute safe levels have not been scientifically established, most guidelines and policies regarding inhaled anesthetics are more advisory rather than mandatory [44].

To control the efficiency of WAG removal, WAG levels in exposure areas should be routinely measured. In the USA, in a survey among USA anesthesiologists, 97% of them reported using anesthesia machines with scavenging systems, showing a great adherence to the NIOSH recommendation [45]. However, not only the recommendations from countries may vary, but also access to scavenging and/or exhaustion systems. These factors can thereby diminish adherence and lead to the exposure of healthcare workers to WAGs [46]. A Brazilian study measured WAG levels in operating rooms with and without scavenging systems and found that, while in the latter the exposure was significantly higher, WAG exceeded the value of 2 ppm in both situations.

### 3.2. Prevention of Exposure to WAGs

The WAG source includes leakage from the anesthesia machine and system, leakage in the breathing system, refilling of vaporizers, and exhalation by patients after the anesthetic procedure has ended [47]. Indoor pollution with WAGs is determined by the anesthesia technique in use, the anesthesia workstations, and/or lack or insufficiency of scavenging systems [48]. By far the worst indoor pollution occurs with open systems where there is no evacuation of the waste gases and no recirculation [47].

Another source of leakage may involve the use of a laryngeal mask or unsealed tracheal intubation. The use of a laryngeal mask in the induction of anesthesia is accompanied by anesthesiologists being exposed to higher levels of the inhaled anesthetic in use [48]. In addition, a ventilation system with turbulent flow is associated with higher exposure to inhaled anesthetics compared to laminar flow [49]. Furthermore, after intravenous induction of anesthesia, depending on the personal preference of the treating anesthetist, mask ventilation with volatile anesthesia is often performed before placing the laryngeal mask/endotracheal tube. Ventilation usually involves a high FGF and an unsealed airway, which can cause considerable indoor contamination.

Pediatric anesthesia is one of the procedures with the highest risk of exposure to inhaled anesthetics [50]. The fast and safe characteristics of inhaled anesthetics to induce anesthesia, together with the advantage of promoting anesthesia without the need for needle puncture in kids, makes it a common and attractive option for pediatric anesthesia [51]. A national-wide survey in Belgium, revealed that the use of gas scavenging during induction is infrequent and it is related to the age of the patient, with systems without scavenging being more used in patients under 1-year-old [51]. During the maintenance of anesthesia, the use of a scavenging system is two-times higher compared with the induction. Likewise, the reduced use of scavenging system in pediatric anesthesia has also been reported in the UK [52]. However, in operating rooms equipped with modern ventilation and scavenging systems, even during pediatric surgery using N_2_O or sevoflurane surgeons were exposed to values of WAG within the recommended limits [53].

Precautionary practices in the administration of inhaled anesthetics vary between pediatric and adult patients, with better prevention of exposure to WAGs during adult surgeries [45]. In cardiopulmonary bypass surgery, for instance, the exposure to sevoflurane generally did not exceed the recommended levels when adequate ventilation and scavenging is available [54,55].

Although several studies have explored WAG levels in the operating rooms, less attention has been given to the post-anesthesia care unit (PACU). Investigation in the PACU of an American hospital confirmed that healthcare workers are exposed to WAGs above the suggested 2 ppm during the 1-h recovery time [56]. However, the use of an appropriate mask in patients of this PACU effectively diminishes exposure to WAG [56]. In contrast, in a German hospital with controlled air exchange systems, only a low trace amount of sevoflurane was measured, with all levels under the limit of 2 ppm [57]. In a pediatric PACU, the recommended WAG levels were also exceeded during the working day, and exposure was related to the number of patients in the recovery room [58]. Even when the construction of the PACU was in accordance with the standards, the environmental WAG level was still higher than recommended.

Currently, the interest in the use of inhaled anesthetics in the intensive care unit (ICU), as an alternative for patient sedation, is growing [59]. The clinical advantages include shortened wakening and extubating times [60]. There are novel devices that have been added to classical ICU ventilators to administer inhaled anesthetics as sedatives, and thus a separate anesthesia machine is not necessary [59]. The effective collection and evacuation of the pollution to the outside atmosphere effectively ensures that the level of indoor contamination during the use of these new technologies remains below the recommended levels [61,62]. Still, careful planning of scavenging and ventilation systems is crucial to prevent excessive occupational exposure.

### 3.3. Health Risks Related to Inhaled Anesthetics Occupational Exposure

The first report of a possible harmful effect of occupational exposure to inhaled anesthetics was in 1967, where surgeons and anesthetists reported headaches and fatigue after a working day using ether [63]. In addition, a high incidence of spontaneous abortion was reported in the interviewed female anesthesiologists [63]. Since then, discussions have commenced regarding the health safety of healthcare professionals involved with inhaled anesthetics.

The potential harm of inhaled anesthetics to reproductive health became a topic of many epidemiologic studies in the early 1970s [64]. A paramount survey performed with almost 50,000 operating room workers showed an increased risk for spontaneous abortion, teratogenic effects, cancer, and hepatic and renal disease in female workers [63]. Remarkably, the same teratogenic effects were also valid for the wives of the exposed male workers [64]. At that time, the most commonly used inhaled anesthetics included N_2_O, halothane, and ether [47]. In the subsequent years, anesthetics such as chloroform, ether, and halothane have been removed due to toxicity [2]. Currently, the oldest inhaled anesthetic still in use is N_2_O. In animal models, N_2_O has a teratogenic effect [65]. However, in clinical settings, the impact of N_2_O and other anesthetic gases in general is less obvious. While the first epidemiological studies from the early seventies reported spontaneous abortion and teratogenic effects due to occupational exposure [63,64], a subsequent scientific review rejected these conclusions based on methodological errors [66]. Even now, controversy remains due to conflicting results. A meta-analysis reported an association between inhaled anesthetics exposure in nurses and an increased risk of poor pregnancy outcomes [67]. On the other hand, a large survey among female doctors from the UK showed that anesthesiologists do not have higher rates of infertility when compared to other specialties that are not exposed to inhaled anesthetics [68].

It is important to emphasize that most of the studies on the subject were performed before scavenging systems were used and with inhaled anesthetics that are no longer in use. Therefore, the potential effects of chronic exposure to inhaled anesthetics regarding reproductive health might be even weaker [69]. However, based on the current literature, the impact of reproductive occupational and also other clinical health risks have not yet been proven [47,70].

Human biomonitoring (HBM) is an important tool to assess human exposure to exogenous substances such as chemical compounds and pollutants by measuring particular compounds or breakdown substances in material collected from subjects exposed to a determined risk agent [71,72]. Different sources of human material can be analyzed to evaluate the toxicity of an agent, including blood, buccal cells, and urine. In the anesthesia field, HBM has already been employed to evaluate the potential risks of occupational exposure to inhaled anesthetics, however, results have so far been inconsistent. There was a significant increase in chromosomal damage in medical staff exposed to inhaled anesthetics when compared with medical staff not exposed [73]. Another study investigated DNA lesions in operating room personnel and there was no significant difference when compared with the healthy population, except for a tendency to accumulate DNA lesions found only in anesthesiologists [74]. However, the authors considered the genotoxic effect to be very weak since there was no massive induction of DNA breaks in the exposed population. On the other hand, a different study demonstrated that WAGs induce sister chromatid changes, a phenomenon that is compared to the same genetic damage as smoking 11–20 cigarettes per day [75].

A systematic review of biomonitoring studies in the operating room personnel concluded that healthcare workers exposed to inhaled anesthetics are at a risk for a cumulative genotoxic effect [76]. In accordance, a meta-analysis of DNA and chromosomal damage based on lymphocyte assays also demonstrated that individuals exposed to inhaled anesthetics show more genotoxic damage when compared with non-exposed individuals [77]. Conversely, a high level of occupational exposure to inhaled anesthetics was associated with genotoxicity in the micronucleus assay, whereas a low level was not [78]. The levels considered as low were within the range of the recommended threshold values from NIOSH, confirming its safety. In agreement, a systematic review found no evidence of adverse effects of inhaled anesthetics when exposure levels were kept within the recommended threshold levels [79].

Notably, a study that included the operating room personnel and PACU workers reported a similar genotoxicity risk for both exposed groups. The risks were significantly different than the non-exposed controls [80]. An additional study compared exfoliated buccal cells from anesthesiologists exposed to inhaled anesthetics for at least 2 years with age and sex-matched internal medicine doctors who were not exposed. The authors reported genomic instability, cytotoxicity, and proliferative risks in the samples from exposed individuals [80]. These findings have shown an increased risk in developing genetic alterations due to the occupational exposure to inhaled anesthetics [81].

The mechanisms involved in genotoxicity and exposure to inhaled anesthetics are still not clear. It is hypothesized that a multi-factorial model including genetic susceptibility, environmental exposure, and individual characteristics such as age, gender, and smoking factors contributes to the development of a disease that is caused by a genotoxic effect [82].

The genotoxic effects of each anesthetic were summarized in an interesting review [82]. All inhaled anesthetics caused genotoxic effects, except for xenon (although there was limited data). Investigations of intravenous anesthetics have shown no genotoxic effect from short-term use. In clinical settings, the influence of a single anesthetic is difficult to be studied, and the literature remains inconclusive. One factor proposed to be involved in the toxicity of inhaled anesthetics is oxidative stress [83]. Indeed, there was a correlation between N_2_O levels in the workspace and oxidative DNA damage, suggesting that increased oxidative stress may be the link between chronic exposure to N_2_O and DNA damage [83]. In addition, systemic inflammation was also evaluated and an increase in interleukin 8 (IL-8) in healthcare workers exposed for 3 years to anesthetic gases was found when compared with non-exposed healthcare workers [84]. Notably, even healthcare workers exposed for a shorter time presented this increased IL-8 expression. Importantly, in this study, the isoflurane, sevoflurane, and N_2_O levels measured in the operating rooms were higher than NIOSH standards [84].

In summary, conflicting data with regards to the health risks of exposure to inhaled anesthetics limit the conclusions to define safety levels or appropriate exposure policies. In addition, biomonitoring studies are generally performed on a small scale and exposure to inhaled anesthetics is normally not distinguished per anesthetic. The current used inhaled anesthetics such as desflurane and sevoflurane are less studied compared with others such as halothane. However, designing and performing a large-scale study to analyze each inhaled anesthetic separately would be extremely difficult due to the heterogeneity of anesthesia protocols and the particularities of workplaces. Therefore, biomonitoring occupational risks due to inhaled anesthetics will probably remain an internal task for each service.

### 3.4. Minimizing Exposure to WAGs

Considering the potential health risks of exposure to WAGs, an obvious policy is to minimize this exposure in healthcare professionals. Different approaches to the protection of workers may be used.

First and most obvious, avoid the use of inhaled anesthetics whenever possible and substitute it for other anesthetic routes (intravenous anesthesia or locoregional techniques). This approach would eliminate any occupational concern related to WAGs. The European legislation regarding hydrofluorocarbon gases states that it should be banned in all situations where a less harmful alternative is possible [85]. Even though inhaled anesthetics are considered of medical need and granted an exception to the rule, if the clinical use of inhaled anesthetics would only be maintained in situations where no alternatives are available or where advantages of the use are expected, there would certainly be a considerable reduction in general use and consequently, healthcare workers’ exposure to WAGs.

Second, installing and maintaining an appropriate scavenge and ventilation system in operating rooms and PACUs. Investing in better and more modern scavenging systems should be encouraged. In addition, protection devices such as special masks may also contribute to the control of WAGs. The use of ISO-Gard masks in patients during anesthetic recovery in the PACU effectively reduced environmental exposure in healthcare workers [86].

Third, controlling WAG levels in services where inhaled anesthetics are used should become routine to ensure that exposure to inhaled anesthetics is within the recommended limit, thereby guaranteeing a safe workplace for healthcare professionals. In addition, this information can augment the current knowledge regarding occupational risks and improve the current recommendations and regulations.

Fourth, improve anesthesia workers’ awareness regarding occupational risks related to their functions to consequently increase adherence and recognition of safety measurements. A pilot study with anesthesiologists revealed a lack of knowledge in major topics of occupational health [87]. In a specific survey on inhaled anesthetics with anesthesiologists and nurse anesthetists, 76% of the participants related to feeling exposed to inhaled anesthetics, however, simple measures for the avoidance of WAGs were not being used [88].

## 4. Inhaled Anesthetics in the Clinical Context

### 4.1. Side Effects

Inhaled anesthetics are known to be much more associated with postoperative nausea and vomiting (PONV) when compared with TIVA and therefore recognized as an independent predictor of PONV [89]. Therefore, omission of volatile anesthetics in favor of propofol is explicitly included in the guidelines for PONV prevention [90].

Malignant hyperthermia is a rare but severe condition that can occur in genetically susceptible patients as a reaction to volatile anesthetics [91]. Patients with malignant hyperthermia present clinical signs such as severe sudden hyperthermia, tachycardia, tachypnea, and acidosis, etc. [92]. To prevent malignant hyperthermia, the use of the intravenous anesthetic propofol is the preferred choice in patients susceptible to the condition [93].

Epileptiform electroencephalogram (EEG) patterns have been associated with the use of sevoflurane for anesthesia induction in both adults and pediatric populations [94]. Risk factors associated with this outcome include female sex, short delay to the onset of anesthesia and the concentration of sevoflurane in use [94,95].

Anesthesia and surgical procedures have been associated with cognitive disorders [96]. Postoperative cognitive disorder (POCD) can impact the affected patients clinically and socially, especially at an older age [97]. Although the causes and risks for POCD are not yet fully understood, the type of anesthesia used—TIVA or inhaled anesthetic—has been suggested to play a role and even if still controversial, the currently available data favor TIVA in place of inhaled anesthesia [97,98,99,100,101].

### 4.2. Anesthetic Conditioning

Inhaled anesthetics are attributed cardioprotection, renal protection, and liver protection, as well as an immunomodulatory effect [102,103,104,105]. Volatile anesthetics seem to activate several of the intracellular effects responsible for ischemic preconditioning, where a short period of ischemia protects most organs, such as the heart, against a subsequent, more severe ischemia [106]. While in vitro studies have yielded seemingly convincing results, the clinical outcomes remain controversial [107].

### 4.3. Cardiac Surgery

Cardiac surgery often requires a period of cardiac ischemia, resulting in a significant incidence of postoperative infarction. Experimental models suggested cardioprotective effects by inhaled anesthetics, similar to ischemic preconditioning [108]. Since then, a variety of studies have investigated the postulated cardioprotective effect of volatile anesthetics in experimental and clinical settings.

In vitro studies and animal models have convincingly shown a cardioprotective effect from volatile anesthetics [109,110]. However, the translation of these positive results to the clinical setting remains an open question [107]. Clinical studies on cardiac surgery have varied from biomarker assessments evaluating the influence of volatile anesthetics in preventing cardiac damage (e.g., via troponin I) to those evaluating clinical outcomes such as cardiac events, length of stay in the hospital, and mortality when compared to the use of TIVA. Different studies have demonstrated the beneficial effect of inhaled anesthetics with regards to biomarker levels [111].

For long-term outcomes, encouraging results have been published in recent years: Lower mortality risks for volatile anesthetics compared with TIVA [112,113]. However, other groups did not obtain the same results [113,114]. Further studies and meta-analysis also demonstrated contradictory results [115,116,117,118].

Finally, a recent large multicenter randomized controlled trial showed that there was no significant difference in 1-year mortality or adverse events between the group receiving volatile anesthetic conditioning when compared to the group with TIVA during cardiac surgery [119]. However, specific characteristics of the study may have influenced the negative result such as the lack of a pre-defined protocol for anesthetic conditioning and the inclusion of patients subjected to on and off pump surgery.

Consequently, after more than 30 years of extensive research, no superiority of either agent can be recommended.

### 4.4. Non-Cardiac Surgery

The potential cardioprotective effects of inhaled anesthetics have also been studied in non-cardiac surgery with a high risk of peri-operative cardiac events [120,121]. Initially, a systematic review and metanalysis about the cardioprotective effect of volatile anesthetics in non-cardiac surgery found a lack of evidence regarding myocardial infarction and mortality and called for more research in the field [122]. Later studies did not show a difference in postoperative cardiac events or mortality with the use of inhalational or non-inhalational anesthesia [114,116,123]. The first randomized controlled trial on the topic also concluded that there was no difference regarding cardioprotection between the use of volatile or intravenous anesthetics [124].

The first study to clinically demonstrate the cardioprotective effect of sevoflurane was performed in patients with coronary artery disease undergoing vascular surgery [125]. Although an encouraging result, this study was considered small and underpowered to make conclusions favoring the use of inhaled anesthetics in non-cardiac surgery [126]. Recently, two different retrospective analyses also failed to show the cardioprotective effect of volatile anesthetics in non-cardiac surgery [127,128].

Unlike the alleged organ protective effects of volatile anesthetics, a study examined N_2_O with regards to its potentially detrimental effects. At first, the use of N_2_O was associated with increased long-term risk of myocardial infarction in patients undergoing non-cardiac surgery, but the same conclusion was not valid for death or stroke [129]. Nevertheless, this study received much criticism due to its design. A second clinical trial was performed and there was no evidence that N_2_O increased the risk of cardiovascular complications or death in major non-cardiac surgery [130]. This finding supported the safety profile of N_2_O [131]. Nevertheless, the use of N_2_O in clinical practice has dramatically declined, mainly due to PONV risk and environmental considerations [132].

### 4.5. Renal Transplantation

A potential protective effect of volatile anesthetics has also been investigated in renal transplantation. In animal models, the use of volatile anesthetics had already been shown to be protective [133]. Therefore, in a clinical setting, it was hypothesized that the use of sevoflurane during a living donor renal transplantation would reduce kidney injury measured by specific biomarkers when compared with propofol-based anesthesia [134]. However, there were comparable results in both groups but a lower acute rejection rate in the sevoflurane group was found 2 years post-transplant [134]. This was suggested since the living donor transplantation might not present sufficient kidney injury to benefit from being “rescued” by volatile anesthetics. Likewise, in another trial comparing sevoflurane versus propofol anesthesia in kidney transplantation, the hemodynamic profile during the surgery as well as postoperative outcomes and complications showed no significant differences between both groups [135].

### 4.6. Lung Surgery

Recent evidence has also brought to light the potential use of inhaled anesthetics for lung protection. Unlike the conditioning effects of volatile anesthetics via attenuation of ischemia-reperfusion injury, the lung protection mechanism seems to be related to an immunomodulatory effect from volatile anesthetics. A prospective study using a large cohort demonstrated that the use of inhaled anesthetics is associated with a dose-dependent decreased risk of early postoperative respiratory complications, mortality, and hospital care costs in patients undergoing non-cardiac surgery [136]. Fittingly, the use of sevoflurane was reportedly associated with a significant reduction in inflammatory markers and better clinical outcomes when compared with the use of TIVA during thoracic surgery with one-lung ventilation [137]. However, in a randomized clinical trial on the same topic, there was no difference regarding major complications when comparing the use of volatile anesthetics versus TIVA [104]. Although the latest results are interesting and intriguing, no conclusion or clinical advice can be provided on the topic based on the current scientific evidence [138].

### 4.7. Cancer Surgery

For solid cancers, surgical removal of the tumor is most of the time the primary treatment and best chance of a cure [139]. Paradoxically, perioperative factors such as the cancer surgery itself and the type of utilized anesthesia may increase the risk of cancer recurrence and mortality [140].

Following the initial experimental models demonstrating that anesthetics could influence cell proliferation and metastasis [141], many basic researchers have tried to unravel the effects of volatile and intravenous anesthetics in cancer progression. Many showed that inhalational anesthetics were associated with direct damage DNA in lung cells and associated with increased risk of metastasis in preclinical models [142,143,144,145]. Likewise, TIVA was associated with anti-cancer properties [146]. Nevertheless, the translation from these results to clinical settings has still not been proven.

Retrospective research on the topic have been controversial. Some studies could show a better survival associated with TIVA in cancer surgery [147,148,149,150], whereas others reported no influence of the anesthesia type on clinical outcomes [151,152,153]. Clinical trials are still ongoing and, therefore, a proper answer to the question is still lacking [154].

A speculative explanation for the conflicting results lays in the hypothesis that a beneficial effect of TIVA might be related to the scale of the surgery, where patients undergoing major cancer surgery would be more likely to benefit from the use of TIVA [155]. This emphasizes the complexity of the subject but if clinical trials prove the superiority of one anesthesia technique, adapting anesthetic practices would potentially be a fast and affordable way of improving postoperative cancer outcomes [156].

### 4.8. Pediatric Surgery

Inhalational anesthesia is traditionally the most commonly used technique in pediatric surgery. However, advances in intravenous anesthesia over the last decades have led to a growing role for its use.

Advantages of inhalational anesthetics include easy induction and no need for intravenous access [157]. Furthermore, inhalational anesthetics can be conveniently managed by the anesthetists. Other potential benefits of inhalational anesthesia, such as cardioprotection in adults have not been confirmed in a pediatric population [158]. On the other hand, the use of TIVA has been associated with reduced complications such as lower incidence of delirium, lower incidence of bronchospasm, and a more peaceful recovery [159,160,161]. Furthermore, volatile anesthesia was more associated with the risk of PONV, whereas different studies have shown a potential antiemetic property of propofol, which is currently considered in guidelines as the indicated anesthesia technique to minimize the risk of PONV [162,163,164].

## 5. Discussion and Conclusions

Climate change is considered the biggest threat to public health in this century. The negative impact of inhaled anesthetics on the environment should be minimized. Different approaches to reduce GHG emissions by anesthesiologists can be briefly summarized: Stop or avoid the use of N_2_O and desflurane, consider the use of intravenous or local-regional anesthesia, and invest in the development of new technologies in scavenging systems.

The occupational risks of exposure to inhaled anesthetics for healthcare professionals are still a matter of concern in anesthesiology. Although the most important health risks such as abortion were associated with inhaled anesthetics no longer in use, the concern related to long-term exposure is ongoing and warrants more regulatory involvement. Recommended exposure limits to the different inhaled anesthetics differ extensively among countries, and there is currently no specific global guideline on the topic. The available data on occupational exposure to inhaled anesthetics are still controversial, but potential genotoxic and carcinogenic effects cannot be excluded. WAG control measures should be implemented as a precaution. Furthermore, increasing awareness of healthcare workers regarding occupational risks, encouraging the reduction of the use of inhaled anesthetics, and investing in appropriate scavenging and ventilation systems are necessary towards a safe work environment.

Although it may seem unlikely that a relatively short intervention such as anesthesia could impact long-term outcomes after surgery, a growing body of data suggest a considerable impact of the anesthesia type. Considering the side effects of anesthesia, inhaled anesthetics increase the risk of PONV. Furthermore, the current literature correlates inhaled anesthesia with POCD, favoring TIVA. In cardiac surgery, while the beneficial use of volatile anesthetics was convincingly shown in experimental and clinical models using biomarkers, trials using clinical outcomes have not confirmed their superiority. Moreover, in noncardiac surgery, there is no definitive evidence for a cardioprotective effect of volatile anesthetics. Furthermore, a potential lung and kidney protective effect has been suggested and is currently under investigation. In cancer surgery, volatile anesthetics seem harmful rather than beneficial, but conflicting data in both experimental and clinical studies limit any definitive conclusion. Finally, in pediatric surgery, TIVA has shown some clinical advantages, and it is expected that its use will grow in the future.

**Figure 2:**
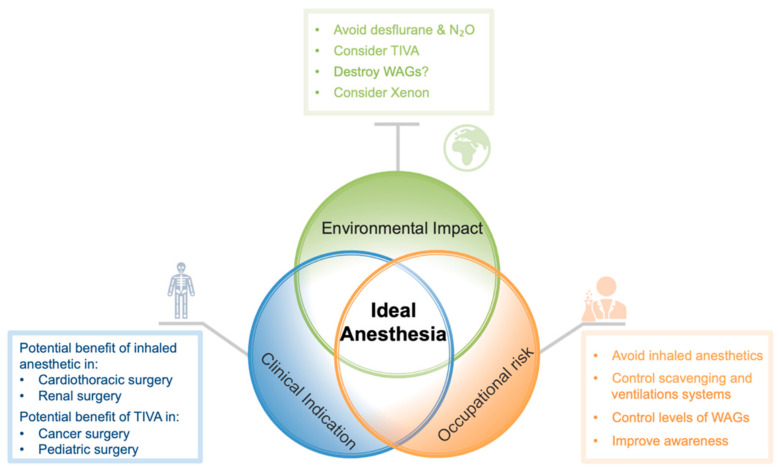
Schematic view of the three different perspectives in choosing an anesthesia technique. Abbreviations: N_2_O: Nitrous oxide; TIVA: Total intravenous anesthesia; WAG: Waste anesthetic gas.

The ideal decision-making process of choosing an anesthesia technique should include the three different perspectives represented in this review (Figure 2). Nevertheless, with the current environmental situation, a drastic reduction in the atmospheric emissions of inhaled anesthetics is necessary and urgent. Consequently, a new approach to the decision-making process of choosing an anesthesia technique should start with the question: Is the use of inhaled anesthetics strictly necessary or potentially clinically better?

Even though the clinical impact of TIVA or inhalational anesthesia is similar or often even superior for TIVA, inhalational anesthesia is still much more frequently used. In the United Kingdom (UK), a survey from the National Health System including all their services showed that 92% of all surgeries are maintained with inhalational anesthesia, with sevoflurane being the most popular volatile anesthetic in use (58.5%) [165]. A survey with Australian anesthetists showed that only 18% of the respondents are frequent users of TIVA [166]. Possible explanations for the low use of TIVA were investigated via another survey, where only 16% of the respondents were considered frequent TIVA users [167]. From the infrequent TIVA users, 52% perceived it as an “additional effort” or justified the infrequent use with other reasons including “difficult for intravenous (IV) access”, “institutional preference”, “lack of real-time monitoring of propofol concentration”, and “risk of missing drug delivery failure” [167]. This result indicates that there are no strong reasons against the use of TIVA and that using inhaled anesthetics might represent conservation of old habits since it has been in use for a longer time.

The idea of completely abandoning the use of inhaled anesthetics due to its impact on the environment has already been proposed [168]. The authors suggested that there is no absolute indication for the use of inhalational anesthesia, hence, it could be replaced by local-regional anesthesia or TIVA. Furthermore, challenges in using TIVA or loco-regional anesthesia are mostly related to the operator confidence in performing it rather than to proper clinical evidence [168]. To overcome these obstacles, a recent guideline has been published with instructions for a safe practice of TIVA [169]. However, it is important to emphasize the lack of appropriate education regarding the environmental impacts and occupational risks of the use of inhaled anesthetics. Taken together, special attention should be given to the educational training in anesthesia as an opportunity to change practices and improve anesthesia from all perspectives.

In conclusion, the use of inhaled anesthetics should be minimized as much as possible to reduce the carbon footprint of anesthesia and consequently protect public health. Still, the choice of a particular volatile anesthetic has much more impact than its elimination. Since replacing desflurane by sevoflurane already eliminates 96% of the greenhouse effect [27], it is essential to emphasize that when a volatile anesthetic is preferred, elimination of the use of desflurane and N_2_O should be encouraged more than converting to TIVA, as this is much easier to accomplish. When clinically indicated (strict indications or potential benefits), workplace conditions should be adequate and healthcare professionals should avoid exposure. The use of inhaled anesthetics or TIVA seems to be comparable in terms of long-term clinical outcomes in different surgery types and TIVA is generally associated with less adverse effects of general anesthesia.

## Data Availability

Not applicable.
